# JACMP 2010–2014

**DOI:** 10.1002/acm2.14449

**Published:** 2024-06-19

**Authors:** George Starkschall

**Affiliations:** ^1^ Department of Radiation Physics University of Texas MD Anderson Cancer Center Houston Texas USA

## 1 INTRODUCTION

The period 2010−2014, during which I served as Editor‐in‐Chief of the JACMP, was characterized by significant growth in the size of the journal. In this article, we examine the growth of the JACMP, both from the point of view of topic coverage and of the source of manuscripts.

## 2 MATERIALS AND METHODS

We examined entries in the URL https://aapm.onlinelibrary.wiley.com/loi/15269914. This URL is a browser that enables access to all back issues of the JACMP. To determine the magnitude of the increase in the size of the JACMP, we examined the following quantities:

1. The number of manuscripts and number of pages in each volume.
2. The categories that held the largest numbers of manuscripts, that is, Radiation Oncology Physics, Medical Imaging, and Radiation Measurements. Other topics, such as Radiation Protection and Regulations, Professional, and Education had too few manuscripts to establish meaningful statistics.
3. We further subdivided Radiation Oncology Physics in more fine‐grained categories and examined trends in the following subcategories: brachytherapy treatment planning, external‐beam treatment planning, dosimetry, tumor localization, treatment delivery, stereotactic radiosurgery, proton treatment delivery, quality assurance, and tumor motion. In some cases, a manuscript could fit into more than one category. In that case an arbitrary decision by the author placed the manuscript in one or the other category.
4. Finally, we compared the country of origin of manuscripts for the time period under study and the most recent issue of the JACMP. In situations in which authors came from different countries, the country of record was taken to be the country of the first author of the manuscript.

## 3 RESULTS

The results of each assessment are found in the following tables:

1. The numbers of manuscripts, total number of pages, and mean length of a manuscript are illustrated in Table 1. We see in this table that during the period from 2010 to 2014 the number of manuscripts published more than doubled, with a corresponding increase in the number of pages. The mean manuscript length, however, remained approximately the same.
2. Although the growth in the JACMP was significant, it was not uniform over subject areas. Table 2 indicates the number of manuscripts and number of pages for the topic Radiation Oncology physics, Medical Imaging, and Radiation Measurements. From this table we see that the greatest contribution to the increased number of manuscripts and number of pages is the increase in number of radiation oncology physics manuscripts. The increases in numbers of manuscripts on other topics was significant as well, but not as pronounced as the increase in number of manuscripts and number of pages devoted to radiation oncology physics (Tables 3 and 4).
3. There does not appear to be any discernable trend in the distribution of topics.
4. A significant change in the JACMP has been the growth in the number of countries from which manuscripts have been published. During the period 2010−2014 the number of manuscripts per year from the USA has increased from 45 to 60, but the percentage of manuscripts from the USA has decreased from 66% to 45%. Between 2014 and 2023 the number of manuscripts has further increased, but the percentage of manuscripts from the USA has remained the same. Moreover, the number of countries from which manuscripts have been published has increased from 12 in 2010 to 23 in 2014 to 27 in 2023.

**TABLE 1:** Number of manuscripts, number of pages, and mean manuscript length of papers published in the JACMP from 2010 to 2014.

Year	Volume	Number manuscripts	Number pages	Mean manuscript length
2010	11	85	1036	12.2
2011	12	100	1156	11.6
2012	13	118	1397	11.8
2013	14	132	1612	12.2
2014	15	173	1952	11.3
% growth (2010–2014)		104%	88%	

**TABLE 2:** Number of manuscripts and number of pages for manuscripts in Radiation Oncology Physics, Medical Imaging, and Radiation Measurements published in the JACMP from 2010 to 2014.

		Radiation Onc physics		Medical imaging		Radiation measurements	
Year	Volume	Number manuscripts	Number pages	Number manuscripts	Number pages	Number manuscripts	Number pages
2010	11	57	728	11	143	8	93
2011	12	82	944	12	151	6	61
2012	13	94	1136	11	127	12	125
2013	14	114	1405	8	95	5	73
2014	15	132	1550	17	178	14	153
% growth (2010–2014)		132%	113%	55%	24%	75%	65%

**TABLE 3:** Distribution of topics of manuscripts in Radiation Oncology Physics published in the JACMP from 2010 to 2014.

	2010		2011		2012		2013		2014	
Topic	Number	% of issue	Number	% of issue	Number	% of issue	Number	% of issue	Number	% of issue
**Brachytherapy treatment planning**	7	11.5%	7	9.2%	7	7.9%	6	5.5%	8	6.5%
**External‐beam treatment planning**	12	19.7%	15	19.7%	20	22.5%	33	30.0%	27	22.0%
**Dosimetry**	8	13.1%	6	7.9%	11	12.4%	9	8.2%	15	12.2%
**Tumor localization**	7	11.5%	4	5.3%	7	7.9%	1	0.9%	2	1.6%
**Treatment delivery**	9	14.8%	12	15.8%	16	18.0%	18	16.4%	20	16.3%
**Stereotactic**	10	16.4%	9	11.8%	6	6.7%	9	8.2%	15	12.2%
**Proton treatment delivery**	3	4.9%	3	3.9%	5	5.6%	1	0.9%	3	2.4%
**Quality assurance**	4	6.6%	14	18.4%	11	12.4%	14	12.7%	23	18.7%
**Tumor motion**	1	1.6%	6	7.9%	6	6.7%	19	17.3%	10	8.1%

**TABLE 4:** Distribution of countries of origin of manuscripts published in the JACMP from 2010 to the present.

	Volume 11		Volume 15		Volume 24	
Country	# Manuscripts	% Manuscripts	# Manuscripts	% Manuscripts	# Manuscripts	% Manuscripts
**USA**	45	66%	60	45%	90	45%
**Australia**	0	0%	4	3%	3	1%
**Austria**	1	1%	0	0%	1	0%
**Belarus**	0	0%	1	1%	0	0%
**Belgium**	0	0%	3	2%	3	1%
**Brazil**	1	1%	1	1%	1	0%
**Canada**	8	12%	9	7%	9	4%
**China**	0	0%	7	5%	20	10%
**Egypt**	1	1%	0	0%	0	0%
**Finland**	0	0%	2	2%	2	1%
**France**	0	0%	0	0%	3	1%
**Germany**	1	1%	5	4%	6	3%
**Greece**	0	0%	0	0%	7	3%
**India**	5	7%	4	3%	0	0%
**Iran**	1	1%	1	1%	4	2%
**Ireland**	0	0%	4	3%	2	1%
**Israel**	1	1%	0	0%	0	0%
**Italy**	1	1%	4	3%	4	2%
**Japan**	2	3%	7	5%	25	12%
**Korea**	0	0%	4	3%	1	0%
**Malaysia**	0	0%	1	1%	0	0%
**Netherlands**	0	0%	2	2%	4	2%
**New Zealand**	0	0%	1	1%	0	0%
**Norway**	0	0%	0	0%	1	0%
**Russia**	0	0%	0	0%	1	0%
**Saudi Arabia**	0	0%	1	1%	0	0%
**South Africa**	0	0%	0	0%	2	1%
**Spain**	0	0%	3	2%	3	1%
**Sudan**	0	0%	0	0%	2	1%
**Sweden**	1	1%	2	2%	2	1%
**Switzerland**	0	0%	2	2%	1	0%
**Syria**	0	0%	0	0%	1	0%
**Taiwan**	0	0%	0	0%	3	1%
**Thailand**	0	0%	0	0%	1	0%
**United Kingdom**	0	0%	5	4%	0	0%
**total**	68		133		202	
**% USA**	66%		45%		45%	
**%USA + %Canada**	78%		52%		49%	
**# countries**	12		23		27	

## 4 CONCLUSION

The JACMP has seen significant growth in the period 2010−2014, growth that has continued to the present. One factor contributing to this growth is an increase in journal submissions in radiation oncology physics. This growth appears to be uniform across subtopics in radiation oncology physics. The second factor contributing to the growth of the journal is the number of foreign submissions. The fraction of manuscripts from the USA has decreased by a third in the 5‐year period from 2010 to 2014. It may be useful in planning for the future of the JACMP to examine the manuscript publication record for the most recent five‐year period (2019‐2023) to determine if the trends observed for 2010−2014 are continuing.

